# Development of Dual ARV-825 and Nintedanib-Loaded PEGylated Nano-Liposomes for Synergistic Efficacy in Vemurafnib-Resistant Melanoma

**DOI:** 10.3390/pharmaceutics13071005

**Published:** 2021-07-01

**Authors:** Yige Fu, Aishwarya Saraswat, Zenghui Wei, Manas Yogendra Agrawal, Vikas V. Dukhande, Sandra E. Reznik, Ketan Patel

**Affiliations:** College of Pharmacy and Health Sciences, St. John’s University, Queens, NY 11439, USA; yige.fu15@stjohns.edu (Y.F.); aishwarya.saraswat19@stjohns.edu (A.S.); zenghui.wei12@my.stjohns.edu (Z.W.); manas.agrawal19@my.stjohns.edu (M.Y.A.); dukhandv@stjohns.edu (V.V.D.); rezniks@stjohns.edu (S.E.R.)

**Keywords:** ARV-825, proteolysis targeting chimera, nintedanib, vemurafenib-resistant melanoma, PEGylated nanoliposomes, synergistic interaction, combination therapy

## Abstract

A novel treatment strategy by co-targeting c-Myc and tumor stroma was explored in vemurafenib-resistant melanoma. BRD4 proteolysis targeting chimera (ARV-825) and nintedanib co-loaded PEGylated nanoliposomes (ARNIPL) were developed to incorporate a synergistic cytotoxic ratio. Both the molecules have extremely poor aqueous solubility. A modified hydration method with citric acid was used to improve the loading of both the molecules in liposomes. ARNIPL with mean particle size 111.1 ± 6.55 nm exhibited more than 90% encapsulation efficiency for both the drugs and was found to be physically stable for a month at 4 °C. Both the molecules and ARNIPL showed significantly higher cytotoxicity, apoptosis and down-regulation of target proteins BRD4 and c-Myc in vemurafenib-resistant cell line (A375R). Vasculogenic mimicry and clonogenic potential of A375R were significantly inhibited by ARNIPL. Tumor growth inhibition in 3D spheroids with reduction of TGF-β1 was observed with ARNIPL treatment. Therefore, ARNIPL could be a promising therapeutic approach for the treatment of vemurafenib-resistant melanoma.

## 1. Introduction

Melanoma is a malignant tumor formed from the melanocyte’s transformation, with an estimated 106,110 new cases to be diagnosed and 7180 people expected to die due to melanoma in 2021 [1]. Genetic analysis of melanoma has allowed us to identify gene mutation in metastatic melanoma, and it was found that 50% of metastatic melanoma patients have BRAF mutation [2]. Vemurafenib and dabrafenib were approved by the FDA as BRAF inhibitors in 2012. Even though targeted therapy provides initial tumor regression, it only offers less than one-year disease control due to the rapid development of resistance [3,4,5,6]. The MEK inhibitor was introduced to be combined with the BRAF inhibitor, which doubles the time to progression due to reactivation of mitogen-activated protein kinase (MAPK) downstream pathway. Nevertheless, cross resistance to MEK inhibitor was also found in cell lines that acquired BRAF resistance, which limits the long-term survival of patients that harbor BRAF mutations [6,7,8]. Considering the limitations of response as well as resistance problems in the current malignant melanoma treatment, novel therapeutic treatment is encouraged to be investigated; especially combining drugs with different mechanisms.

Emerging data have suggested that the pathogenesis of melanoma is due to the aberrant activity of epigenetic regulation of the transcriptional process through the modification of DNA and chromatin, which affects melanoma promotion, metastasis, and drug resistance [9,10]. Overexpressed c-Myc was reported to drive melanoma metastasis by promoting vasculogenic mimicry via c-Myc/snail/Bax signaling, and major resistance pathways were found to converge to activate c-Myc [11,12]. c-Myc is a major transcriptional factor directly regulated by BRD4 and controls almost all cellular processes. However, lack of specific active site makes the direct therapeutic targeting difficult [13]. Indirect targeting of c-Myc by BET bromodomain inhibition has proved as therapeutic strategy in recent years [14,15,16]. It was also reported that BRD4 is significantly up-regulated in primary and metastatic melanoma tissues compared to melanocytes and thus can be considered to be a new target for therapeutic strategy [12]. PROteolysis TArgeting Chimeric (PROTAC) to destroy ‘undruggable’ proteins was discovered in 2001 and was considered to be the next-generation tool for chromatin regulation [17,18]. We previously revealed that a novel PROTAC molecule—ARV-825 (ARV) is very promising in the treatment of vemurafenib-resistant melanoma [19].

One of the great challenges in delivering an anti-cancer drug to tumor cells is the stromal component’s richly dense and hard to penetrate tumor microenvironment, which affects melanoma tumorigenesis including initiation, progression, and metastasis [20,21]. It was reported that melanoma cells can stimulate the recruitment of fibroblasts and activate them, which contributes to melanoma growth as well as drug resistance [22]. Crosstalk was reported between cancer cells and cancer associated fibroblasts (CAFs) remodels the stromal extracellular matrix (ECM) and contributes to the cancer progression. TGF-β1 is one of the major cytokines derived from CAFs and was found to increase survival of human melanoma through stroma remodeling [23]. BRAF inhibitor vemurafenib-treated melanoma cells was found to lead to transforming growth factor beta 1 (TGF-β1) release, which increases the deposition of fibronectin, type I collagen, α-smooth muscle actin ‘as well as CAFs [24,25]. Type I Collagen and fibronectin rich dense ECM of solid tumor serves as a tortuous, viscous, and steric barrier, which severely restricts the uptake and antitumor efficacy of nanotherapeutics [26]. Nintedanib (Ni) is a Food and Drug Administration (FDA) approved anti-fibrotic agent for the treatment of idiopathic pulmonary fibrosis. Ni is a multitarget inhibitor of tyrosine kinases, which showed inhibition of ECM proteins such as fibronectin, type I collagen, and transforming growth factor TGF-β1-induced myofibroblast transdifferentiation, resulting in reducing the dense network in the tumor ECM [27,28]. Thus, Ni was selected here as an anti-fibrotic agent to target tumor stroma by inhibiting TGF-β1-induced fibroblast.

Considering the potential of PROTAC technology in cancer treatment, there is a great need for a formulation for the delivery of ARV. Commercial products demonstrated the potential of stealth or long circulating nanoliposomes in delivery of anticancer drugs [29,30,31,32]. Therefore, PEGylated liposomes could be considered to be the most safe and effective approach for simultaneous delivery of ARV and Ni. The aim of this study was to evaluate the cytotoxic interaction of Ni and ARV-825 in the vemurafenib-resistant melanoma cells, develop ARNIPL, and explore anti-melanoma efficacy of ARNIPL. We hypothesize that anti-fibrotic Ni would enhance the penetration of nanocarrier into tumors by remodeling of tumor stroma. Combined targeting of c-Myc via BRD4 degradation and stromal component with translational nanocarrier will be effective in treatment of vemurafenib-resistant melanoma.

## 2. Materials and Methods

### 2.1. Materials

ARV-825 was obtained from ChemieTek (Indianapolis, IN, USA), Ni and Vemurafenib was purchased from LC Laboratories (Woburn, MA, USA), 1,2-Dioleoyl-*sn*-glycero-3 phosphocholine (DOPC) was purchased from Cordenpharma (Liestal, Switzerland), PE 18:0/18:0-PEG2000 was obtained from Lipoid (Ludwigshafen, Germany), Cholesterol and Chloroform were purchased from Sigma-Aldrich (St. Louis, MO, USA), The TGF-β1 ELISA kits were purchased from Invitrogen (Carlsbad, CA, USA). Other chemicals and materials were described in Supplementary Materials.

### 2.2. Analytical Method

The analytical method using HPLC for ARV and Ni detection are described in detail in the Supplementary Materials.

### 2.3. Cell Viability Assay and Effect of Drug Combination

The cytotoxicity of ARV, Ni and ARNIPL were evaluated in A375R and SK-MEL-28R using 3-(4,5-dimethylthiazol-2-yl)-2,5-diphenyl tetrazolium bromide (MTT) assay and the combination index (CI) were calculated as described in detail in the Supplementary Materials.

### 2.4. Preparation and Characterization of ARNIPL

The preparation of ARNIPL using modified hydration method and was described in the Supplementary Materials [33]. The average size, size distribution by intensity, zeta potential of ARNIPL were measured using dynamic light scattering (DLS) particle size analyzer (Malvern Zetasizer Nano ZS, Royston, UK). Samples were analyzed using disposable cuvettes at 25 °C with a scattering angle of 173°. Amicon ultra centrifugal filters (50 K) were used to analyze the entrapment efficiency of ARNIPL. The sample of total and free drug were collected, and the concentration was analyzed by HPLC. The encapsulation efficiency was calculated using the following formula:

Percent encapsulated = ([Total drug] − [Free drug])/[Total drug] × 100%(1)

### 2.5. In Vitro Release Study

The release of ARV and Ni from ARNIPL was carried out using the dialysis bag method as described in detail in the Supplementary Materials.

### 2.6. Enzyme Linked Immunosorbent Assay

The enzyme linked immunosorbent assay (ELISA) kit was used to determine the level of TGF-β1 secreted by A375, SK-MEL-28, A375R and SK-MEL-28R and 3D spheroids on the day 6 after various treatments. The method is described in detail in the Supplementary Materials.

### 2.7. Western Blot Assay

The effect of Ni, ARV and ARNIPL on protein expression of BRD4, BCL-2 and c-Myc were evaluated by Western blot assay. The detail methods were described in the Supplementary Materials.

### 2.8. Clonogenic Assay

The ability of clonogenicity inhibition of A375R for various treatments were evaluated by clonogenic assay as was described in the Supplementary Materials.

### 2.9. Vasculogenic Mimicry

A375R cells suspension at 2 × 10^5^/mL were incubated with ARV (0.2 μM), Ni (0.7 μM) and ARNIPL (ARV 0.2 μM and Ni 0.7 μM) for 5 min at 37 °C followed by seeding in a 96 well plate precoated with 50 μL BME at a density of 2 × 10^4^/well. After 24 h incubation at 37 °C with 5% CO_2_, images were taken using an EVOS light microscope at 20×. The number of branching points were quantified for tube formation.

### 2.10. Flow Cytometry for Apoptosis Analysis

A375R were seeded at a density of 1 × 10^5^/mL in 6-well plate and cells were treated with ARV (1 μM), Ni (3.5 μM), ARNIPL (contains ARV 1 μM and 3.5 μM Ni) for 24 h incubation. Then cells were collected by centrifugation at 3000 rpm for 5 min and resuspended with DMEM media (contains 1% bovine serum albumin and 1% FBS) to a concentration of 5 × 10^5^ cells/mL. Apoptosis analysis was carried out by Muse Annexin V & Dead Cell Assay kit (Millipore Sigma, St. Louis, MO, USA). Briefly, the cell suspension was diluted in 1:1 ratio with MUSE Annexin V dead cell reagent, followed by incubated for 20 min at room temperature, then the samples were analyzed for apoptosis using Muse^®^ Cell Analyzer (Millipore Sigma, St. Louis, MO, USA).

### 2.11. Spheroids Development and Characterization of 3D Multicellular Tumor Spheroids

Tumor spheroid of A375R alone and co-culture of A375R+ dermal fibroblast (1:0.5) were prepared as follow; Briefly, cells were seeded at a density of 1500 cells/well in ultra-low attachment treated spheroid microplate (Corning Life Sciences, St Lowell, MA, USA). The plate was centrifuged at 150× *g* for 10 min and incubated overnight. The cells were then treated with ARV, Ni, ARNIPL and ARV + Ni with 1 μM ARV and 3.5 μM Ni in each group. Media was added as a control. The media was replaced with fresh treatment every alternative day until day 6. Moreover, same treatment groups with higher concentration (2 μM ARV and 7 μM Ni) were also investigated in co-culture spheroids and treated until day 4. Images of 3D spheroids were taken at 20× magnification every time before treatment using EVOS^®^ FL Auto Imaging System (Thermo Fisher Scientific, Waltham, MA, USA). The cell viability and 3D cell imaging were taken on day 7 of treatment as described in the Supplementary Materials.

### 2.12. Statistics

Statistics were carried out using GraphPad Prism7 Software (GraphPad, La Jolla, CA, USA). Each experiment has been performed in triplicate and the data were reported as the mean ± standard deviation (SD). Statistical analysis was performed by Student’s *t*-test, one-way ANOVA-Bonferroni’s or two-way ANOVA followed by Tukey-Kramer post-hoc multiple comparison test. *p* < 0.05 was considered to be statistically significant.

## 3. Results

### 3.1. Enzyme Linked Immunosorbent Assay

**Figure 1:**
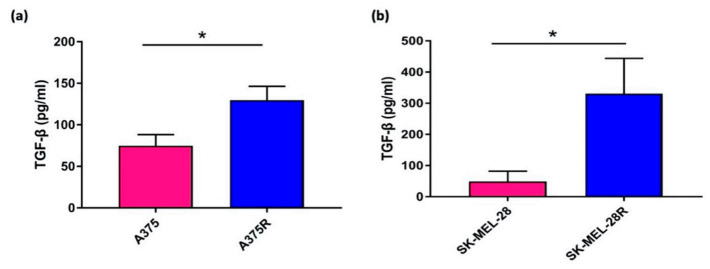
ELISA analysis of TGF-β1 produced by BRAF^V600E^ mutated parent and vemurafenib-resistant melanoma cell lines. Results are expressed as the amount (pg/mL) of TGF-β1 produced by the same number of cells of (**a**) A375 and A375R, (**b**) SK-MEL-28 and SK-MEL-28R (* *p* < 0.05).

To investigate whether TGF-β1 production is more in the vemurafenib-resistant melanoma cells than BRAF^V600E^ mutated melanoma cell lines, two BRAF^V600E^ mutated melanoma cell lines A375 and SK-MEL-28 and their vemurafenib-resistant cells lines were used in ELISA assay to compare the amount of TGF-β1 release from the same number of cells. The result shown in Figure 1 revealed that a significant increasing amount TGF-β1 was found in the vemurafenib-resistant cell lines, which suggested the potential of targeting TGF-β1 in vemurafenib-resistant melanoma.

### 3.2. Cell Viability Assay and Effect of Drug Combination

**Figure 2:**
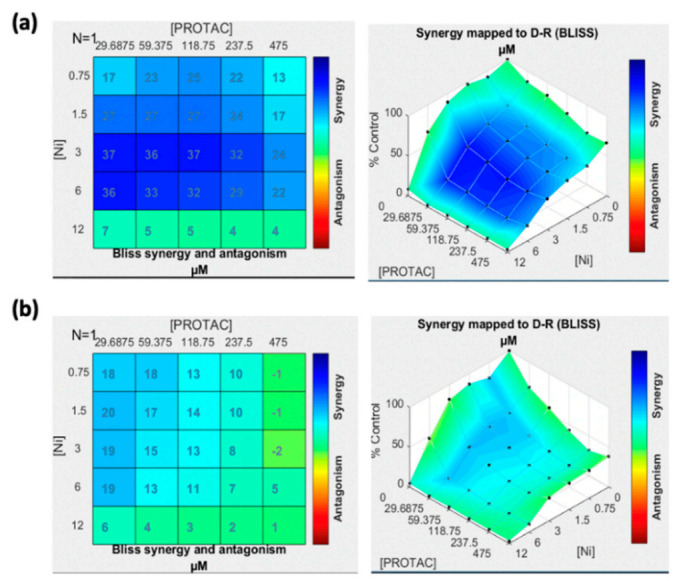
Combenefit mapped surface output for the drug combinations involving ARV and Ni using Bliss synergy model. ARV and Ni synergistically inhibit cell growth in a panel of (**a**) A375R and (**b**) SK-MEL-28R. Cells were treated with ARV and Ni in a 5 × 5 concentration grid for 48 h, cell viability was determined by MTT assay. The darker the blue color, the more predicted synergy between the drugs (*n* = 3).

**Figure 3:**
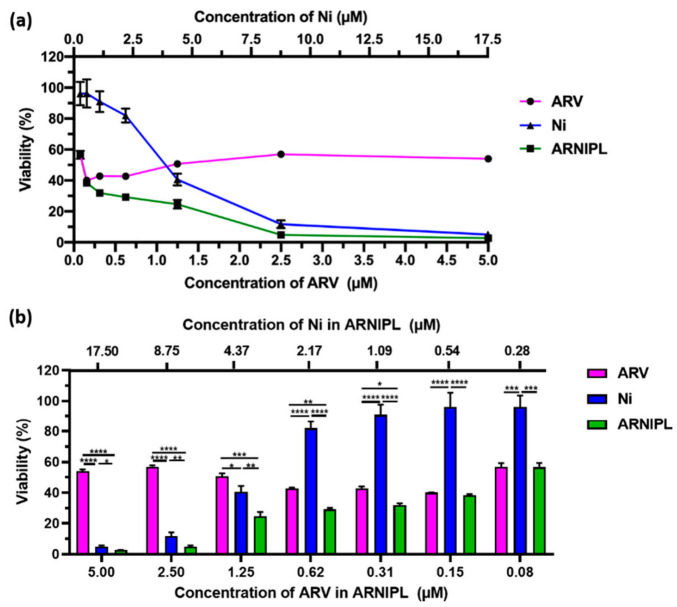
Cytotoxicity assay of ARV, Ni and ARNIPL in A375R. (**a**) % Cell viability with ARV, Ni and ARNIPL treatment in A375R. (**b**) The % viability comparison of ARV, Ni and ARNIPL at various concentrations. Data were plotted as mean ± SEM (*n* = 3). (* *p* < 0.05, ** *p* < 0.01, *** *p* < 0.001, **** *p* < 0.0001).

The effect of molecule combination was analyzed using Combenefit software. The contour plot of synergy/antagonism with the Bliss model is shown in Figure 2. The positive scores mean the drug combination are synergistic while the negative scores indicated the combination was antagonist. All positive scores were observed in A375R while in SK-MEL-28R, the scores are less with lighter blue color. The result suggested that the synergistic effect of ARV and Ni was stronger in A375R compared with SK-MEL-28R. Thus, further anti-cancer efficacy studies of ARNIPL were evaluated in A375R. As shown in Figure 3, free Ni and ARNIPL killed cells in dose-dependent manners. However, ARV did not show further killing above 1 μM. At low very concentration, ARV was found to dominate the killing in the ARNIPL while Ni did not show too much killing. Nevertheless, with increase in concentration, Ni showed promising killing of melanoma cells and the combination with ARV in ARNIPL further decreased the viability. Additionally, the IC_50_ of ARV and Ni in the liposomes were lower than the free drug (Table 1). The calculated combination index (CI) of ARV and Ni was 0.54 ± 0.05 while the CI of ARNIPL showed 0.59 ± 0.12, indicating there is synergism between Ni and ARV and the synergism remains similar in ARNIPL.

### 3.3. Characterization and Stability of ARNIPL

Due to the poor entrapment efficiency (EE) and drug loading (DL) of ARV and Ni, citric acid was incorporated in the hydration step to enhance the EE and DL via interaction of citric acid with basic drugs. Initially, 1% ARV and 2% *w*/*w* Ni were loaded into the liposomes, the EE without critic acid of ARV and Ni were 79.68%, 21.67% respectively, while result in more than 90% EE of both of the drugs with the citric acid. Moreover, the DL of ARV and Ni was also increased with citric acid (Table 2). Thereafter, we prepared batches with high DL and high concentration of ARV and Ni. The particle size and zeta potential of optimized ARNIPL are shown in Supplementary Figure S1, the mean particle size of ARNIPL (optimized) is 111.1 ± 6.97 nm, which is in the range of enhanced and permeation (EPR) effect that allows particles to easily extravasate into tumors [27,34]. The polydispersity index was less than 0.3, which indicates the particles were homogenous distributed. The zeta potential of ARNIPL (optimized) was found to be +13.9 ± 6.62, which may mainly attribute to the orientation of basic (amine group) toward surface of ARNIPL (optimized) with lipophilic part entangled in lipid bilayer. Physical stability of ARNIPL (optimized) prepared was analyzed after a month storage at 4 °C. The particle size of ARNIPL (optimized) was 111.5 ± 6.55 with polymer dispersity index less than 0.25 and zeta potential was found to be 12.1 ± 5.61 mV (Supplementary Figure S2a,b). Moreover, ARNIPL (optimized) was found to be physical stable for one month at 4 °C storage (Supplementary Figure S2c), which indicated the ARNIPL (optimized) was stable after a month storage.

### 3.4. In Vitro Release Study

**Figure 4:**
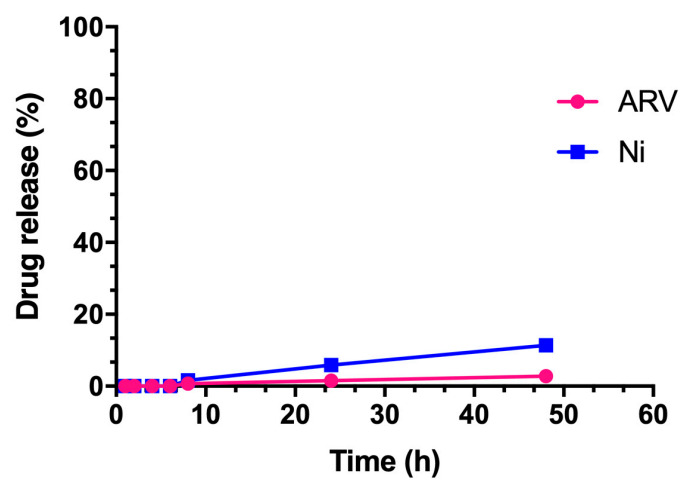
In vitro release study of ARNIPL. Release of ARV and Ni were observed at pH 7.4 in sink condition.

The result showed less than 2% of ARV and less than 5% of Ni was released in 24 h. After 48 h, the percentage release of both drugs increased but was within 5% for ARV and less than 10% for Ni at sink conditions (Figure 4), which indicated that ARNIPL did not show any burst release of ARV and Ni.

### 3.5. Clonogenic Assay

**Figure 5:**
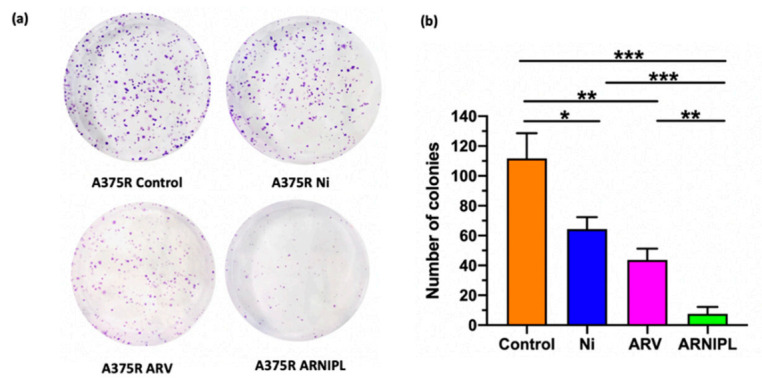
Colony forming ability of A375R after treatment with ARV, Ni and ARNIPL (**a**) Crystal violet staining images of A375R after various treatments. (**b**) Number of colonies with ARV, Ni and ARNIPL treatment and control in A375R. Number of colonies with ARNIPL treatment were significantly reduced compared to other groups (* *p* < 0.05, ** *p* < 0.01, *** *p* < 0.001).

The ability to form colonies after treatment was analyzed by clonogenic assay, which also determines cell reproductive death after treatment. Results as shown in Figure 5 suggest that the number of colonies were significantly reduced by the exposure to Ni and ARV alone group (* *p* < 0.05, ** *p* < 0.01). ARNIPIL showed 8–20 folds lesser number of colonies compared to drug alone and control group. Plating efficiency (PE) of A375R control was 40%. Survival fraction (SF) of ARNIPL was much lower compared to other treatment groups as shown in Table 3.

### 3.6. Vasculogenic Mimicry

**Figure 6:**
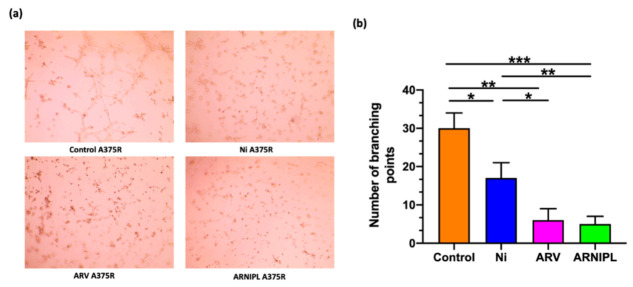
Evaluating the effect of ARNIPL on A375R vasculogenic mimicry (**a**) Vasculogenic mimicry images of A375R treated with ARV (0.2 μM), Ni (0.7 μM) and ARNIPL (ARV 0.2 μM and Ni 0.7 μM) (**b**) Number of branching points after treatment with ARV, Ni and ARNIPL treatment in A375R. (* *p* < 0.05, ** *p* < 0.01, *** *p* < 0.001).

Melanoma vasculogenic mimicry was first described and characterized by Maniotis’ group, where the tube formation was distinct from endothelial cells [35]. The formation of vasculogenic mimicry was observed in A375R on the Matrigel. ARV and Ni inhibited vasculogenic mimicry at very low concentration as shown in Figure 6a. ARNIPL containing ARV and Ni showed further inhibition of vasculogenic mimicry compared to each individual drug. The number of branching points are plotted in Figure 6b, where ARV and ARNIPL both showed most significantly lower number of branching points (** *p* < 0.01, *** *p* < 0.001). There was no statistically significant difference between ARV and ARNIPL.

### 3.7. Western Blot Assay

**Figure 7:**
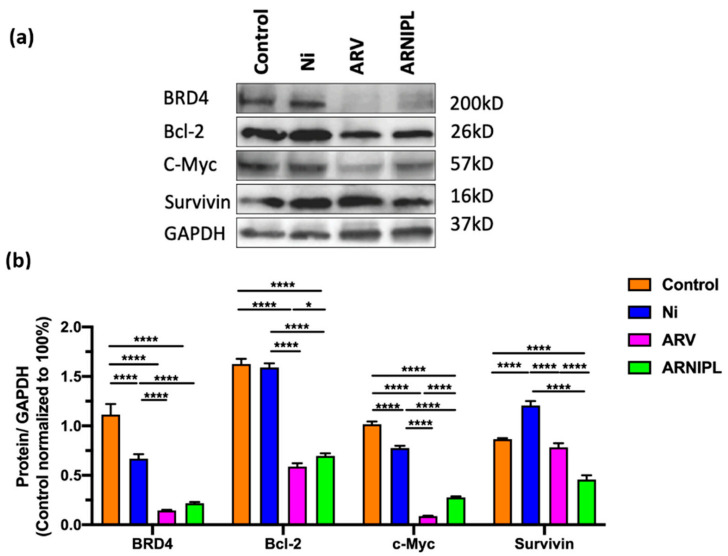
Western blot analysis results. (**a**) Results of expression of apoptotic proteins were determined by Western blot assay after 24 h treatment. (**b**) Quantitation of the Western blot results. Higher apoptotic protein expression was observed in ARV and ARNIPL treated A375R cells when normalized to GAPDH (*n* = 3). (* *p* < 0.05, **** *p* < 0.0001).

The protein expression of BRD4, BCL-2 and c-Myc was significantly lower the whole cell lysates from A375R with ARV and ARNIPL treatment while increased amount of Survivin was observed (Figure 7). Furthermore, the expression of antiapoptotic protein was lower in ARV and ARNIPL groups and cleaved caspase-3 were found to be significantly higher in treatment groups compared with control, which further confirmed that the apoptosis induced by ARNIPL treatment.

### 3.8. Apoptosis Assay

**Figure 8:**
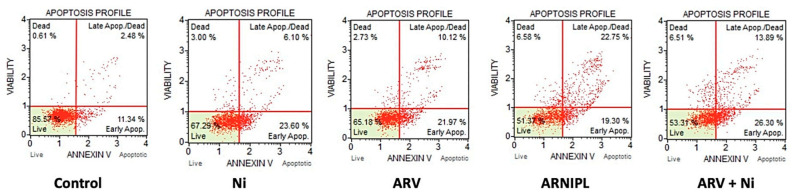
Flow cytometric apoptosis analysis in A375R treated with 3.5 μM Ni, 1 μM ARV, ARNIPL and ARV + Ni (3.5 μM Ni and 1 μM ARV) after 24 h treatment, ARNIPL showed higher apoptotic cell population compared with control.

Apoptosis of vemurafenib-resistant melanoma cell line A375R was carried out by flow cytometry, which shows percentage of early and late apoptosis distribution of the treated cells. The total apoptosis was calculated as the sum of early apoptosis and late apoptosis. Total apoptosis of Ni, ARV, ARNIPL and ARV + Ni is shown in Figure 8, where ARNIPL and the combination of ARV and Ni showed significantly higher amount of apoptosis compared to ARV and Ni alone. As expected, there was no difference in number of apoptotic cells in ARV + Ni (Added from DMSO stock) and ARNIPL (The same concentration was added as in liposomal formulation).

### 3.9. Determination of ARNIPL Efficacy in 3D Tumor Spheroids

#### 3.9.1. Development and Characterization of 3D Tumor Spheroids

**Figure 9:**
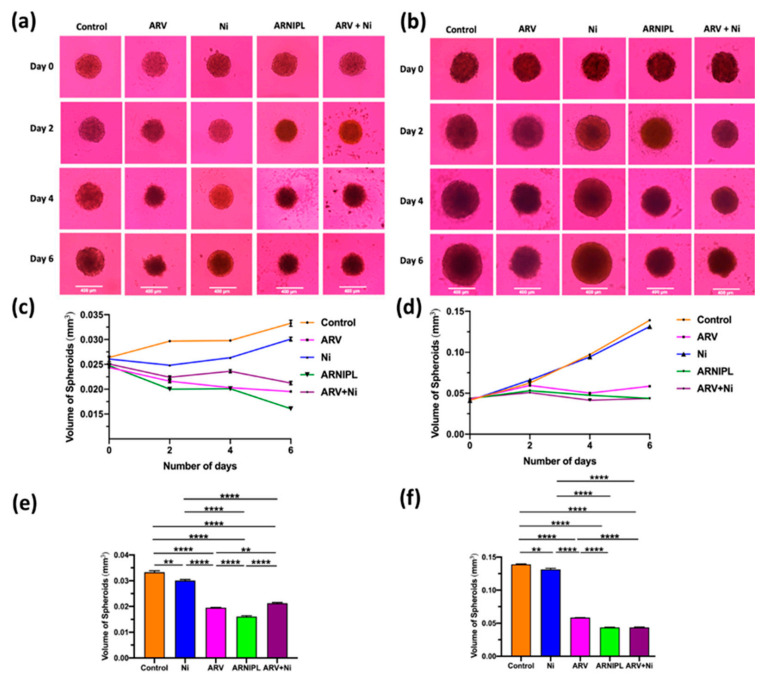
The effect of various treatments on A375R and A375R + Dermal Fibroblast co-culture 3D multicellular tumor spheroids growth. Spheroids were treated with control, 1 μM ARV, 3.5 μM Ni, ARNIPL and ARV + Ni (1 μM ARV and 3.5 μM Ni) (**a**) Bright field images of A375R spheroids with treatments on days 0, 2, 4, and 6. (**b**) Bright field images of co-culture spheroids with treatments on days 0, 2, 4, and 6. (**c**) Comparison of the volume of A375R spheroids. (**d**) Comparison of the volume of co-culture spheroids with various treatments at day 0, 4 and 6. (**e**) The volume of A375R spheroids on day 6. (**f**) The volume of co-culture spheroids on day 6. Significant difference in volume of spheroids was observed with ARV, Ni, ARNIPL and ARV + Ni compared to control. (** *p* < 0.01, **** *p* < 0.0001).

To better mimic in vivo tumor growth, 3D multicellular tumor spheroids of A375R and co-culture spheroids with dermal fibroblasts were developed to evaluate the efficacy of ARNIPL. According to the bright field images of A375R and co-culture spheroids with different treatments on days 0, 2, 4, and 6 as shown in Figure 9a,b, the growth of co-culture spheroids was found to be much faster than the spheroids that only contains A375R. Moreover, the killing pattern of ARV and Ni observed from the surface of the spheroids was different. The killing effect of ARV can be observed on the surface, as seen from the irregular surface of the spheroids on day 6 while Ni treated spheroids showed more intact smooth surface. The surface of combination of spheroids treated with both drugs in ARNIPL and ARV + Ni was uneven. Ni treated groups also showed dark and dense core, which may be due to the apoptotic cells present on the periphery of the spheroids. As for A375R spheroids growth as a function of time (Figure 9c,d), Ni group inhibited the growth of spheroids as compared to the control group until day 6. ARV, ARNIPL and ARV + Ni treated groups however showed a higher and substantial inhibition of tumor growth compared with control. The volume of A375R spheroids with various treatments were compared on day 6 as shown in Figure 9e,f, all the treatment groups showed significant tumor volume reduction compared with control group. Precisely, ARV treated group displayed more reduction of tumor volume than Ni treated group, and the combination of both drugs in ARNIPL and ARV + Ni further decreased the volume of the spheroids. Moreover, ARNIPL treated groups presented lower tumor volume compared with ARV + Ni, which may be due to the better penetration of the liposomes. The reduction of volume with ARV, Ni, ARNIPL and ARV + Ni treatment compared to control were 41.34%, 9.60%, 51.71% and 36.19%, respectively. The volume of the co-culture spheroids with various treatments as a function of time are shown in Figure 9d, spheroids showed rapid growth in control and Ni treated groups while other treatment groups showed significant inhibition in terms of tumor growth. The tumor volume of various treatments was compared at day 6 as shown in Figure 9f where all the groups showed significant tumor inhibition compared with control. On day 6, The reduction of volume with ARV, Ni, ARNIPL and ARV + Ni treatment compared to control are 57.14%, 7.14%, 71.43% and 71.43%, respectively. ARV treated group exhibited more inhibition than Ni treated group in terms of tumor volume. In addition, the combination group of drugs in ARNIPL and ARV + Ni showed further reduction of tumor volume compared to individual drugs. No significant difference in tumor volume was observed in ARNIPL compared to ARV + Ni in 3D co-culture spheroids on day 6.

#### 3.9.2. Enzyme Linked Immunosorbent Assay in 3D Spheroids

Before the treatments on day 6, the supernatant of both A375R and coculture with different treatments were collected for analyzing TGF-β1 secretion. As shown in Supplementary Figure S3, all the treatment groups showed lower amount of TGF-β1 compared to the control on both the types of tumor spheroids. Groups with ARV showed significant reduction in TGF-β1 compared to other groups.

#### 3.9.3. 3D Cell Viability Study

The CellTiter-Glo luminescent cell viability assay was performed to study the number of viable cells in treated A375R and co-culture spheroids on day 7, as shown in Supplementary Figure S4. ARNIPL and ARV + Ni exhibited a significantly reduced number of alive cells compared to control, Ni and ARV. Moreover, Ni also showed decreased cell viability in co-culture spheroids compared to control.

#### 3.9.4. 3D Tumor Spheroid Live and Dead Cell Imaging

Supplementary Figure S5 showed the spheroids treated with ARV, ARNIPL and ARV + Ni had higher red intensity compared to control and Ni group. ARV and ARNIPL treated groups exhibited stronger red fluorescent intensity indicating higher killing of melanoma cells. ARV and ARNIPL treated groups showed strong red intensity representing the dead cells.

## 4. Discussion

To overcome the current problems of targeted therapy such as resistance, relapse and limited efficacious in melanoma patients, we proposed an alternative strategy for the treatment of vemurafenib-resistant melanoma. We are particularly focusing on epigenetic regulator and stromal factors those are implicated in BRAF inhibitor mediated resistance. ARNIPL were successfully developed for the first time to target both epigenetic regulators and stromal components of the malignant melanoma.

Dual-loaded drug liposomes has emerged as an encouraging drug delivery system with promising efficacy in cancer treatment [36,37,38,39]. Advantages of liposomes in terms of long circulation, biocompatibility leads to improved safety, bioavailability and efficacy of drugs that are encapsulated in the phospholipid bilayer [40,41]. Loading of brick dust molecules such as ARV and Ni in liposomes is challenging. Since ARV is poorly soluble in ethanol and ether, traditional method for liposomes preparation such as ethanol or ether injection is not applicable. In this study, we have adopted a modified hydration method and used acid base interaction to enhance the EE and drug loading of both ARV and Ni in the nanoliposomes Due to the basic property of the drugs, we selected citric acid due to its safe use in parenteral delivery. Marketed products such as ZOFRAN^®^ include citric acid monohydrate with the concentration of 0.05% for injection purpose. Hence, citric acid was incorporated in the hydration step of liposomal preparation in order to stabilize the drugs, which showed enhanced EE for both ARV and Ni. Moreover, PEGylation on the surface of liposomes leads to improved stability, enhanced circulation time, avoiding reticuloendothelial system (RES) uptake and enhanced EPR effect after intravenous administration, which is the most common strategy for liposomes delivery [27,42,43].

The combination of FDA-approved anti-fibrotic agent Ni combined with BRD4 PROTAC molecule ARV exhibited synergistic effect in the nanoliposomes using two vemurafenib-resistant cell lines. However, ARV did not show further killing above 1 μM, which can be explained by “hook effect” phenomenon. This phenomenon is attributed to the mechanism of the PROTAC molecule, which tends to form a binary complex with either E3 ligase or protein of interest instead of forming ternary complex at higher concentration [44]. Therefore, the combination of Ni with ARV may not only serve a dual-functional targeting purpose, but also alleviate the limitation of “hook effect”. Moreover, the calculated combination index of ARV + Ni and ARNIPL in A375R indicated that the synergy of ARV and Ni was achieved by incorporating two molecules within the liposomes. Additionally, ARNIPL was found to be stable for a month as liquid form at 4 °C. Still, freeze drying of liquid liposomes would be a good choice to achieve long-term stability. As for release study, no burst release was observed for ARNIPL and there was minimum release of both ARV and Ni for 48 h, which means the drug will stay in the liposomes in the blood circulation while the drugs would release after cellular uptake of liposomes by the tumor cells. Therefore, the side effects that of these drugs would be minimized.

A hetero-bifunctional PROTAC molecule ARV was reported to exhibit faster and more efficient degradation of BRD4, suppression of c-Myc and cell proliferation inhibition compared to small molecule BRD4 inhibitors [45]. Moreover, recent research suggested that ARV is more potent compared to small molecule BRD4 inhibitors OTX015 and JQ1 in the clonogenic assay, which is consistent with our result herein [46]. ARNIPL exhibited more predominant inhibition of melanoma cells to form colonies, which is in accordance with our cytotoxicity suggesting that the combination of drugs exerted synergistic effect in melanoma tumor inhibition. Furthermore, TGF-β1 was found to related with regulating clonogenicity of melanoma cells and TGF-β1 inhibition could block the clonogenicity through SMAD4-independent inhibition of mitosis [47]. Thus, the effect of Ni in clonogenicity assay could related with TGF-β1 pathway. The overexpression of c-Myc was also reported to promote vasculogenic mimicry and melanoma metastasis [11]. Vasculogenic mimicry is a different vascular formation mechanism compared to traditional angiogenesis, which is formed by tumor cells and was related to the poor survival [48]. We previously demonstrated that ARV has promising effect in the inhibition of vasculogenic mimicry in A375R [49]. In the present paper, Ni was also able to inhibit vasculogenic mimicry. This may be due to inhibition of multiple signaling pathway, which was reported to be a potential target for anti-vasculogenic mimicry in cancer [50]. The expression levels of BRD4, c-Myc and Bcl-2 and survivin were analyzed by Western blot, which showed significant reduction of BRD4, c-Myc and Bcl-2 and surviving with ARV and ARNIPL treatment. As for the result of apoptosis assay, ARNIPL and ARV + Ni groups showed higher population of early/late apoptosis compared to single ARV or Ni treatment. The apoptotic effect of ARV was reported as a result of disrupting BRD4 that is expressed in various types of cancer [51,52,53,54,55].

Additionally, vemurafenib led to secretion of many other factors in tumor microenvironment and contribute to the microenvironment-driven resistance to BRAF inhibition due to the dense network in tumor stroma [47]. As a result, it is difficult for nanotherapeutics to penetrate deeply into tumors and release the drug into tumor cells, thus the anti-tumor efficacy would be limited. Targeting stromal factors has been proved as a promising strategy in melanoma since microenvironment not only promote tumor growth, angiogenesis, metastasis, but also contribute to the resistance problem through growth factor signaling modulation [48,49,50]. Nanoparticle therapies have been extensively studied to modify tumor stroma due to its critical role in tumorigenesis [51]. Due to the limitation of two-dimensional (2D) cultures to mimic tumor stroma and the interaction with other types of cells such as fibroblasts, A375R and coculture with fibroblasts 3D spheroids were developed to enhance the biological relevance in investigating the anti-melanoma efficacy of ARNIPL. We found that co-incubation of A375R with dermal fibroblasts significantly promoted growth of A375R spheroid growth. The spheroid growth-promoting effect from fibroblasts co-culture has previously been discussed due to the role of fibroblasts in tumor progression [56]. The reduction of tumor spheroids with Ni and ARV at low concentration suggested the drugs are very potent. Moreover, the killing pattern on the surface of the spheroids indicated the different mechanism of killing for both the drugs. The surface of spheroids with ARV treatment exhibited uneven surface which means ARV inhibits tumor growth by killing melanoma cells from the surface. As for the Ni treatment, the spheroids surface remains in a regular round shape while the tumor growth has been inhibited, which implied the growth inhibition could be related with the regulation of melanoma cells through various signaling pathways. For instance, Ni could inhibit multiple factors and reduce CAFs through TGF-β1 inhibition, which affects the proliferation of melanoma cells. The combination of both drugs in ARNIPL and ARV + Ni showed better inhibition in tumor volume compared to individual drugs, this further confirmed the importance of drug combination and could be related with synergistic effect of ARV and Ni. Significantly higher tumor inhibition in ARNIPL than ARV + Ni could be attributed to better penetration of the liposomes. As for the 3D spheroid imaging in the co-culture model, minimal green fluorescent signal in control group suggested that more aggressive growth of melanoma cells compared to fibroblasts. Surface of control group looks mostly covered with melanoma cells in control group. The fibroblasts have a slower growth rate then cancer cells which has also been reported previously [56]. However, fluorescent signal from fibroblasts can be observed on the surface of the spheroids with Ni treated group, this may be due to Ni inhibit the growth of melanoma cells (at lower concentration) through inhibition of multiple pathways and thus result in slower growth of melanoma cells compared to GFP-fibroblasts. However, further investigation is sought to understand the penetration of free drug molecule vs. liposomes in spheroid environment, mechanism of Ni and ARV on fibroblasts in the 3D spheroids and efficacy of such combination in vivo.

Overall, ARNIPL showed encouraging tumor growth inhibition in the 3D tumor spheroids compared to single ARV and Ni treatment, which suggested the importance of this combination. Moreover, significantly lower amount of TGF-β1 was detected after ARNIPL treatment, which could further inhibit tumor growth that is promoted by CAFs.

## 5. Conclusions

In conclusion, PROTAC molecule ARV and anti-fibrotic agent Ni loaded nanoliposome (ARNIPL) was successfully developed using modified hydration method. To the best of our knowledge, the combination effect of ARV and Ni was investigated for the first time in vemurafenib-resistant melanoma, which exhibited synergistic effect in anti-melanoma efficacy in vitro and strong tumor-suppressive effect in 3D spheroid model. Overall, ARNIPL could provide a promising alternative therapeutic strategy for melanoma patients exhibiting vemurafenib-resistance.

## Figures and Tables

**Table 1:** In vitro cytotoxicity of Ni, ARV alone and in the liposomes in A375R (*n* = 3). Data were shown as mean ± standard deviation (**** *p* < 0.0001).

Drug	Ni	ARV	ARNIPL-Ni	ARNIPL-ARV
**IC_50_ (μM)**	4.35 ± 0.47	0.13 ± 0.08	0.24 ± 0.05 ****	0.07 ± 0.06

**Table 2:** Particle size, zeta potential and entrapment efficiency (EE) of ARNIPL (*n* = 3).

Group	Size	Zeta Potential	EE of ARV (%)	EE of Ni (%)	DL of ARV (%*w*/*w*)	DL of Ni (%*w*/*w*)
**ARNIPL** **(without citric acid)**	138.4 ± 6.66	−25.2 ± 4.41	79.68 ± 7.60	21.67 ± 2.15	0.80 ± 0.08	0.43 ± 0.04
**ARNIPL** **(with citric acid)**	99.62 ± 4.78	−5.34 ± 3.82	94.15 ± 3.48	97.16 ± 2.33	0.94 ± 0.03	1.94 ± 0.05
**ARNIPL** **(optimized)**	111.1 ± 6.55	+13.9 ± 6.62	97.80 ± 3.20	96.86 ± 2.63	1.96 ± 0.05	3.87 ± 0.11

**Table 3:** Clonogenic Assay: Surviving Fraction (SF) of treatment cells (*n* = 3); S.D. = Standard deviation.

%SF ± S.D.	Ni	ARV	ARNIPL
A375R	47.5 ± 4.19	37.5 ± 3.81	18.0 ± 2.25

## Supplementary Materials

The following are available online at https://www.mdpi.com/article/10.3390/pharmaceutics13071005/s1, Figure S1: Dynamic light scattering graphs, Figure S2: Stability result of ARNIPL, Figure S3: In vitro release study of ARNIPL, Figure S4: TGF-β1 levels were analyzed by ELISA and are expressed as the amount (pg/mL) of TGF-β1 produced by A375R and coculture spheroids with various treatments on day 6 (**a**) A375R spheroids (**b**) coculture spheroids, Figure S5: 3D cell viability assay conducted using CellTiter-Glo^®^ kit, Figure S6: 3D spheroid live & dead cell imaging on day 6 in A375R and co-culture 3D spheroids.
