# Supplementary material for: Development of Dual ARV-825 and Nintedanib-Loaded PEGylated Nano-Liposomes for Synergistic Efficacy in Vemurafnib-Resistant Melanoma
Source: Pharmaceutics. 2021 Jul 1;13(7):1005. doi: 10.3390/pharmaceutics13071005 (PMC8308940; doi:10.3390/pharmaceutics13071005)
Supplement: Supplementary file 1 [file pharmaceutics-13-01005-s001.zip › pharmaceutics-1221695-supplementary.pdf]

pharmaceutics

Supplementary Materials: Development of Dual ARV-825 and Nintedanib-Loaded PEGylated Nano-Liposomes for Synergistic Efficacy in Vemurafnib-Resistant Melanoma

Yige Fu, Aishwarya Saraswat, Zenghui Wei, Manas Yogendra Agrawal, Vikas V. Dukhande, Sandra E. Reznik and Ketankumar Patel

Supplementary information

Materials

Dulbecco's modified Eagle's medium (DMEM) was purchased from ThermoFisher Scientific Inc. (Waltham, MA, USA), Fetal Bovine Serum (FBS) was procured from Atlantic Biologics (Oakwood, GA, USA). Penicillin-Streptomycin-Amphotericin B (PSA) was purchased from MP Biomedicals, LLC (Solon, Ohio, USA). 3-(4,5-dimethylthiazol-2-yl)-2,5-diphenyl tetrazolium bromide (MTT) was acquired from Fisher Scientific. Bradford dye reagent was obtained from Alfa Aesar (Haverhill, MA, USA). Other chemicals and solvents were of analytical grade or chromatographic purity. The cell culture methods are described in the Supplementary material.

Methods

Cell culture. Melanoma cell lines (A375 and Sk-Mel-28) were purchased from American Type Culture Collection (Manassas, Virginia, USA). Vemurafenib-resistant melanoma cell lines (A375R and SK-MEL-28R) was developed by adding vemurafenib to the cell line as the method described before [1]. The vemurafenib resistance was confirmed prior to studies. GFP expressing human dermal fibroblasts (HDFCs-adGFP) were obtained from angio-proteomie (Boston, MA, USA). Cells were cultured in DMEM medium supplemented with 10% FBS at 37 °C with 5% CO_2 in humidified incubator.

Analytical Method. Chromatographic separation method of ARV and Ni was developed using Waters e2695 separation module, equipped with 2998 Photo diode array (PDA) detector and Hypersil ODS C18 column (250 mm × 4.6 mm, 5 μm). The mobile phase was Acetonitrile:Potassium dihydrogen phosphate buffer (10 mM) of pH 3.5 (70:30) with a flow rate of 1 mL/min. The temperature of the column was kept at 25 °C and Empower 3 software was used to monitor output signal. ARV was detected at 247 nm and Ni was detected at 390 nm. The retention time of ARV and Ni were 4.48 ± 0.02 min and 9.15 ± 0.36 min, respectively.

Cell viability assay and effect of drug combination. Logarithmic growth phase cells were seeded in 96-well plates at a density of 5000 cells/well. After 24 h, drugs and formulation were diluted in cell culture medium to achieve different concentrations. After 48 h treatment, cells were incubated with 20 μL of 5 mg/mL MTT solution in each well and incubated for 3 h at 37 °C, 5% CO_2. Then the medium was removed, and MTT-formazan crystals were dissolved by adding 100μL of dimethyl sulfoxide (DMSO) to each well. The quantity of MTT-formazan was determined by 570 nm absorbance on an Epoch2 microplate. IC_{50} were calculated using GraphPad Prism7 Software. Effect of ARV and Ni combination was assessed and according to Combination index (Chou-Talalay method) and Combenefit software [2]. Following equation was used to calculating combination index (CI):

$$CI = (D)1/(Dx)1+(D)2/(Dx)2 \quad (1)$$

where (Dx)1, (Dx)2 = the concentration of the tested substance 1 and the tested substance 2 used in the single treatment that was required to decrease the cell number by 50%

and (D)1, (D)2 = the concentration of the tested substance 1 in combination with the concentration of the tested substance 2 that together decreased the cell number by 50%.

Preparation of ARNIPL. Modified hydration method was used for the preparation of ARNIPL. Briefly, ARV:Ni:DOPC:cholesterol:DSPE-PEG2000:citric acid in a 1:3.5:45:15:2 molar ratio was dissolved in chloroform. Parenteral-grade mannitol (200 μm) was used as absorbent and the chloroform solution was drop-wise added to mannitol with constant stirring at 45 °C. The resultant paste was left overnight for chloroform evaporation. The resultant powder was dispersed in water contains citric acid at 55 °C followed by sonication (30% amplitude) for 2 min. The same method was employed for the preparation of liposome without citric acid for comparison.

Western Blot Assay. 2×10^5 of A375R were plated and grown for 24 h. The cells were treated with 350 nM Nintedanib; 100 nM ARV and ARNIPL (350 nM Nintedanib and 100 nM ARV) for 24 h. Cells were then lysed in Modified Radioimmunoprecipitation assay (RIPA) buffer containing N-ethylmaleimide, phosphatase and protease inhibitor. The lysates were normalized for protein concentration using the Bradford Assay. Western blotting was used to measure specific protein expression in sets of four samples: Control (No treatment), Ni, ARV, and ARNIPL. The proteins were separated on 4–20% SDS-PAGE gradient gel (Bio-Rad, CA, USA) and subsequently transferred to polyvinylidene difluoride (PVDF) membrane (Millipore Sigma, MO, USA). The membranes were for 60 min with 5% non-fat milk in TBST (10 mM Tris, pH 8.0, 150 mM NaCl, 0.05% Tween 20) and then incubated with antibodies against BRD4 (1:1000; Cell signaling, Danvers, MA, USA), Bcl-2 (1:1000; Cell signaling, Danvers, MA, USA), c-Myc (1:1000; Cell signaling, Danvers, MA, USA), Survivin (1:1000; Cell signaling, Danvers, MA, USA), and GAPDH (1:5000; Proteintech, Rosemont, IL, USA) at 4 °C for 12–16 h. Membranes were washed three times for 10 min with TBST and incubated with a 1:5000 dilution of horseradish peroxidase-conjugated anti-mouse or anti-rabbit antibodies for 1 h. The membranes were again washed three times with TBST before chemiluminescence imaging on Azure c500 using the ECL, Pico or Femto substrates. Protein band image density was analysed using ImageJ.

Stability study. ARNIPL prepared by modified hydration method were used for the stability study. Particle size, zeta potential, drug content and entrapment efficiency after one month of storage at 4 °C were evaluated. Samples were withdrawn at different time points after centrifugation of ARNIPL at 5000 rpm for 10 min. The concentration of drug at each time points were analyzed using HPLC. The percentage drug content was plotted at different time intervals.

In vitro release study. Dialysis bags (Spectra/Por® 7) were soaked in Milli-Q water at room temperature overnight to remove the preservative. Release of ARV and Ni were carried out at 37 °C in a dialysis sac with 100 mL of phosphate buffer saline (pH 7.4) containing 0.5% TPGS with constant stirring. The samples were withdrawn from the release medium at different time intervals up to 48 h. The concentration of ARV and Ni in the release media was evaluated by HPLC. The percentage of release of each drug was plotted versus time points.

Enzyme linked immunosorbent assay. For 2D culture, cells were seeded at 1.2×10^4 cells per well in a 24-well plate and incubated at 37 °C with 5% CO_2. Cells were harvested when 90% confluency was reached. Then the supernatants were collected by centrifugation at 1000× *g* for 10 min at 4 °C. For 3D spheroid culture, the supernatant in each well was taken on the 6th day before treatment. Three independent experiments were conducted in duplicate according to the manufacture's protocol. Briefly, 100 μL of sample or TGF-β1 standard protein per well was incubated for 2 h at room temperature in ELISA plate that coated with capture antibody. Afterward, the samples were completely washed and 100 μL TGF-β1 detection antibody was added to each well for 1 h incubation followed by incubation with Avidin-HRP and tetramethylbenzidine (TMB) substrate. Finally, after adding stop solution, the optical density (OD) values of different samples were detected at 450 nm by using a microplate reader (Dynex Technologies, Chantilly, VA, USA). The

concentration of TGF-β1 was interpolated using a second-order polynomial (quadratic) equation generated from a standard curve in GraphPad Prism 7.

Clonogenic assay. Clonogenic assay was carried out according to the procedure described previously [3]. A375R were seeded at a density of 1000 cells/well in a 6-well plate. The cells were allowed to attach for around 5 h. Then the cells were treated with ARV (0.2 μM), Ni (0.7 μM) and ARNIPL (ARV 0.2 μM and Ni 0.7 μM) before the population doubling. The next day, the medium was replaced and cells were maintained at 37°C with 5% CO_2 for 5 days. Then cells were rinsed with PBS followed by glutaraldehyde (6.0% *v*/*v*) fixation and 0.5% crystal violet staining for 30 min. Thereafter, the plate was washed with water and left for drying. Colonies were counted (the colony is defined to consist of at least 50 cells) on the following day. Plating efficiency (PE) and survival fraction (SF) was calculated by following equations:

$$\text{PE} = \text{number of colony formed} / \text{number of cells seeded} \times 100\% \quad (2)$$

$$\text{SF} = \text{PE of treated sample} / \text{PE of control} \times 100 \quad (3)$$

3D cell viability study. The viability of 3D spheroids was analyzed using CellTiter-Glo kit (Promega, Madison, WI, USA). After imaging on day 7 of treatments, equal volume of CellTiter-Glo® reagent was added in the well (100 μL of CellTiter-Glo® reagent and 100 μL of cell culture media in each well) and the plate was vigorously shaken for 5 min to induce cell lysis, followed by incubation at room temperature for 25 min to stabilize the luminescent signal. The luminescence was then measured using a Spark 10 M plate reader (Tecan, Männedorf, Switzerland).

3D spheroid cell imaging. On the 7th day of treatment, spheroids were washed twice with phosphate-buffered saline (PBS) followed by staining with 3 μM EthD-1 and 1 μg/mL DAPI (Santa Cruz Biotechnology, Dallas, TX, USA) in PBS solution. The plate was incubated at 37°C for 3 h and fluorescent images were then taken at 20X using EVOS-FL Cell Imaging fluorescence microscope (Thermo Fisher Scientific, Waltham, MA, USA).

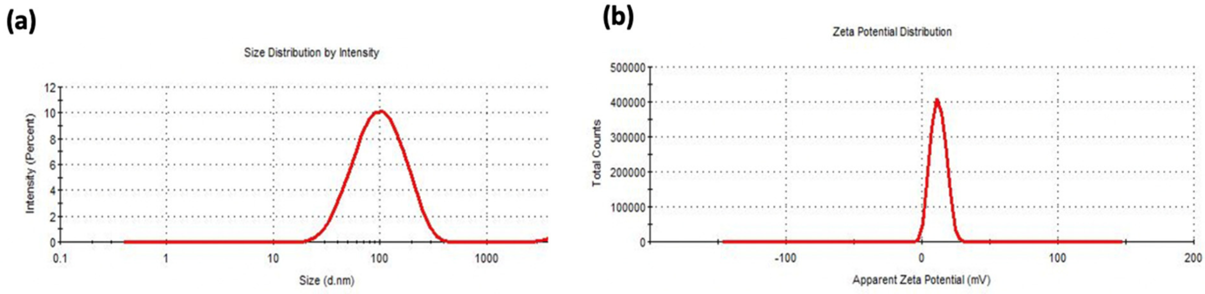

Figure S1. Dynamic light scattering graphs. (**a**) Particle size distribution with an average diameter. (**b**) zeta potential of ARNIPL.

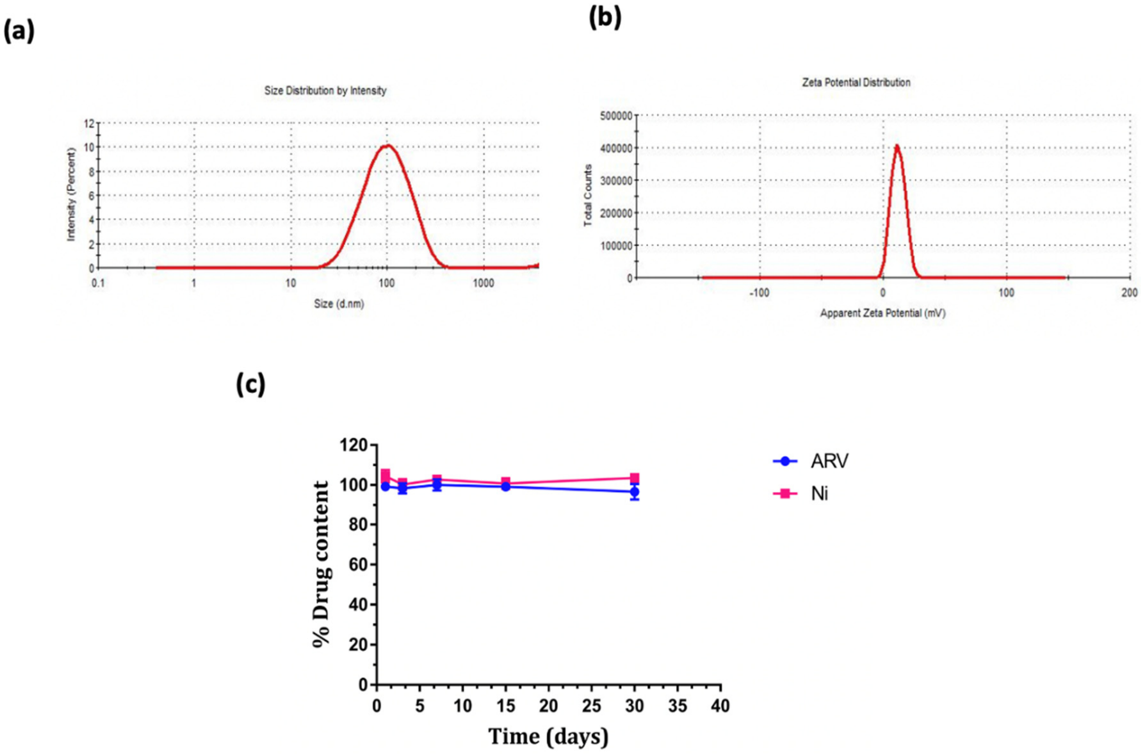

Figure S2. Stability result of ARNIPL. (**a**) Particle size and (**b**) zeta potential of ARNIPL after a month storage at 4 °C. (**c**) Drug content after a month storage at 4 °C.

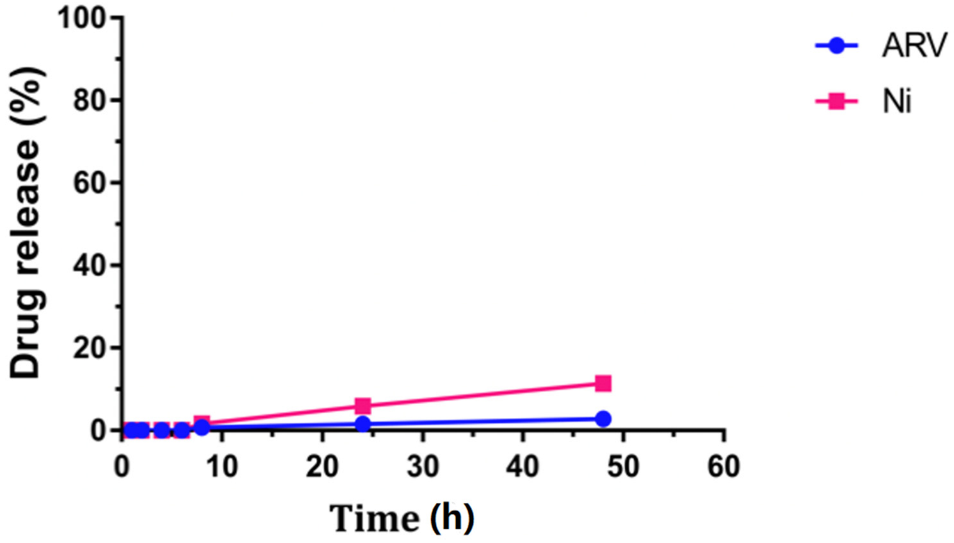

Figure S3. In vitro release study of ARNIPL. Release of ARV and Ni were observed at pH 7.4 in sink condition.

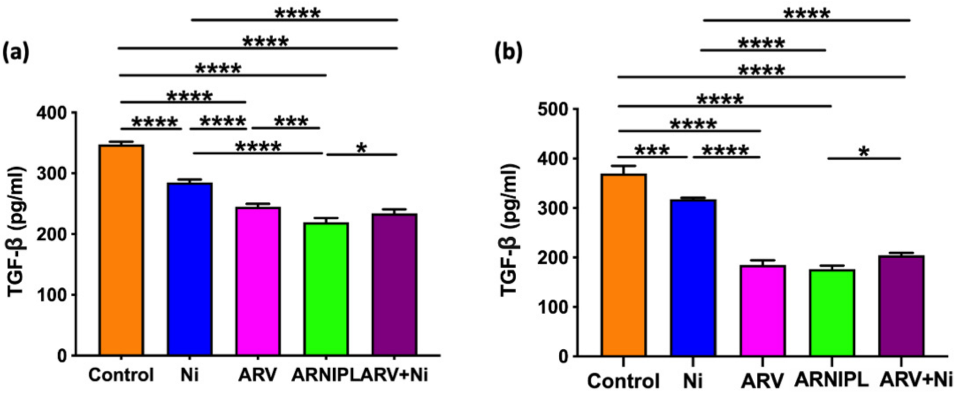

Figure S4. TGF-β1 levels were analyzed by ELISA and are expressed as the amount (pg/mL) of TGF-β1 produced by A375R and coculture spheroids with various treatments on day 6 (**a**) A375R spheroids (**b**) coculture spheroids. Data shown are the means ± SD ($n = 3$). (* $p < 0.05$, *** $p < 0.001$, **** $p < 0.0001$).

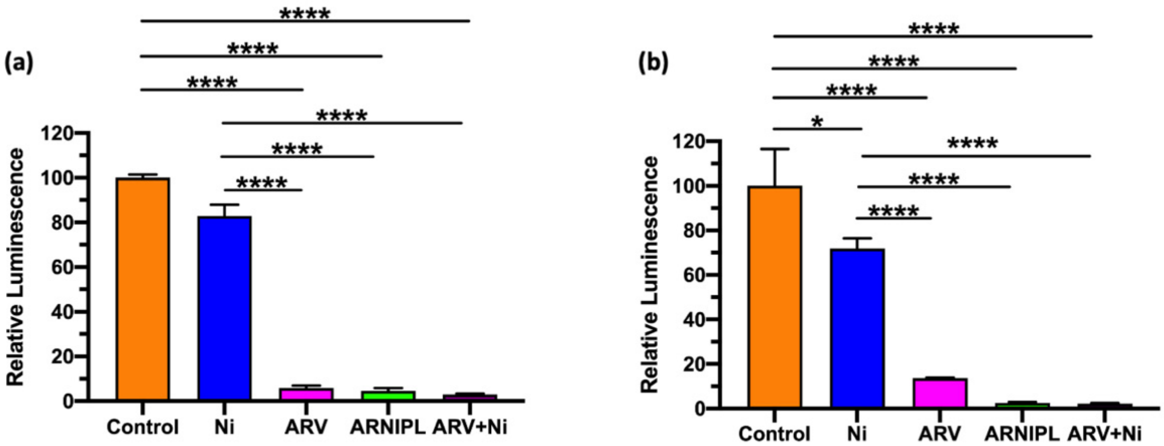

Figure S5. 3D cell viability assay conducted using CellTiter-Glo® kit. Results of various treatments in (**a**) A375R and (**b**) Co-culture 3D spheroids are shown as relative luminescence normalized to control (100%). (* $p < 0.05$, **** $p < 0.0001$).

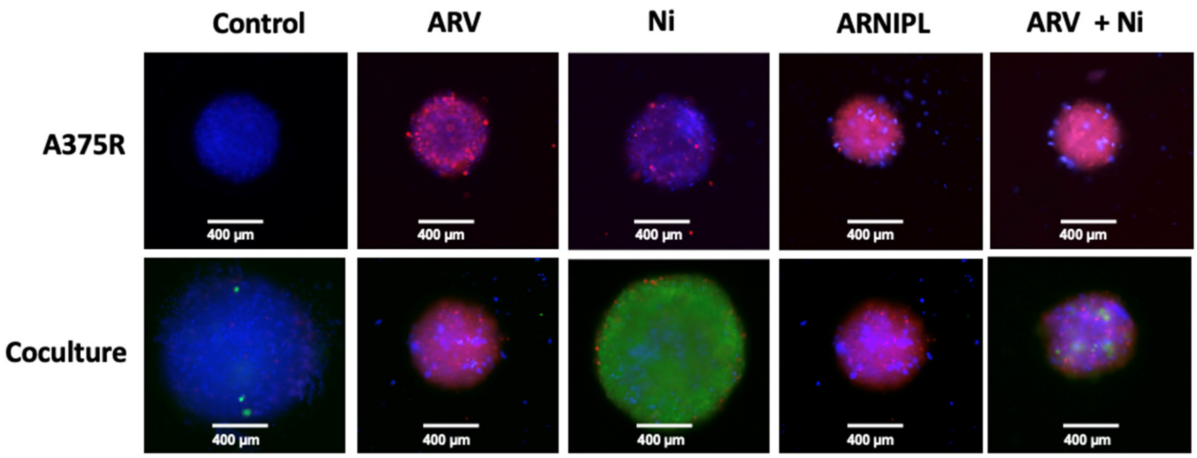

Figure S6. 3D spheroid live & dead cell imaging on day 6 in A375R and co-culture 3D spheroids. Spheroids were stained with DAPI (blue) for nuclei, EthD-1 (red) for compromised/dead cells, green fluorescence was shown by GFP-labeled fibroblasts. Representative images were taken at 20X magnification. The fluorescent images of the dead cells and nuclei were taken on day 7. The red fluorescence obtained from ethidium homodimer-1 represents dead cells in the spheroids while blue color stained by DAPI represent cell nuclei. Green fluorescence was shown by GFP-labeled fibroblasts.

References

1. Rathod, D.; Fu, Y.; Patel, K. BRD4 PROTAC as a novel therapeutic approach for the treatment of vemurafenib resistant melanoma: Preformulation studies, formulation development and in vitro evaluation. *Eur. J. Pharm. Sci.* **2019**, *138*, 105039.
2. Chou, T.-C. Drug combination studies and their synergy quantification using the Chou-Talalay method. *Cancer Res.* **2010**, *70*, 440–446.
3. Franken, N.A.; Rodermond, H.M.; Stap, J.; Haveman, J.; van Bree, C. Clonogenic assay of cells in vitro. *Nat. Protoc.* **2006**, *1*, 2315–2319.
